# Impact of Blunted Perception of Dyspnea on Medical Care Use and Expenditure, and Mortality in Elderly People

**DOI:** 10.3389/fphys.2012.00238

**Published:** 2012-07-04

**Authors:** Satoru Ebihara, Kaijun Niu, Takae Ebihara, Shinichi Kuriyama, Atsushi Hozawa, Kaori Ohmori-Matsuda, Naoki Nakaya, Ryoichi Nagatomi, Hiroyuki Arai, Masahiro Kohzuki, Ichiro Tsuji

**Affiliations:** ^1^Department of Internal Medicine and Rehabilitation Science, Tohoku University Graduate School of MedicineSendai, Japan; ^2^Department of Epidemiology, School of Public Health, Tianjin Medical UniversityTianjin, China; ^3^Department of Geriatrics and Gerontology, Institute of Development, Aging and Cancer, Tohoku UniversitySendai, Japan; ^4^Department of Public Health and Forensic Medicine, Tohoku University Graduate School of MedicineSendai, Japan; ^5^Department of Science in Sports and Exercise, Tohoku University Graduate School of MedicineSendai, Japan

**Keywords:** dyspnea, the elderly, medical cost, medical service use, mortality

## Abstract

Dyspnea is an alarming symptom responsible for millions of patient visits each year. Poor perception of dyspnea might be reasonably attributed to an inappropriately low level of fear and inadequate earlier medical treatment for both patients and physicians, resulting in subsequent intensive care. This study was conducted to evaluate medical care use and cost, and mortality according to the perception of dyspnea in community-dwelling elderly people. We analyzed baseline data from a community-based Comprehensive Geriatric Assessment in 2002. The perception of dyspnea in 479 Japanese community-dwelling elderly people with normal lung function was measured in August 2002. The sensation of dyspnea during breathing with a linear inspiratory resistance of 10, 20, and 30 cmH_2_O/L/s was rated using the Borg scale. According to the perception of dyspnea, we divided the elderly into tertiles and compared all hospitalizations, out-patient visits, costs, and death through computerized linkage with National Health Insurance beneficiaries claims history files between August 2002 and March 2008. In-patient hospitalization days and medical care costs significantly increased with the blunted perception of dyspnea, resulting in an increase in total medical-costs with blunted perception of dyspnea. With low perception group as reference, the hazard ratios of all-cause mortality were 0.65 (95% CI 0.23–1.89) for intermediate perception group and 0.31 (0.10–0.97) for high perception group, indicating the mortality rate also significantly increased with the blunted perception of dyspnea after multivariates adjustment (*p* = 0.04). The blunted perception of dyspnea is related to hospitalization, large medical costs, and all-cause mortality in community-dwelling elderly people. These findings provide a rational for preventing serious illness with careful monitoring of objective conditions in the elderly.

## Introduction

Dyspnea is one of the most common symptoms responsible for millions of patient visits each year, and one of the most common reasons for emergency department visits and hospitalization (Fadullo et al., 1986; Parshall, 1999). Dyspnea is not only an unpleasant physical sensation which causes reductions in functional status and quality of life, but also an important alarming symptom for both patients and doctors, as it is often a harbinger of severe pathology, especially in elderly patients. The awareness that early events portend an increasing severe condition is important for timely treatment.

The etiology of dyspnea is often cardiopulmonary causes such as congestive heart failure, asthma exacerbation, chronic obstructive pulmonary diseases, pneumonia, and pulmonary embolism. However, the other causes of dyspnea, such as sepsis, anemia, acidosis, and neuromuscular diseases, must always be considered (Waseem et al., 2006). Although many of the causes of dyspnea are life-threatening, the symptom appears highly variable in comparison to levels of pathophysiology (Manning and Schwartzstein, 1995). The affective unpleasantness of perceived dyspnea has been suggested as being particularly important for motivating patients to initiate adaptive behavior, such as medication intake and physician visits, in a timely manner (Banzetto et al., 2000). In the elderly, there is a perceptual slowing for symptoms including dyspnea (Tack et al., 1982; Killian et al., 2000). Moreover, the elderly present special difficulties as several causes of dyspnea often coexist, which may further delay correct diagnosis and adequate therapy.

Since dyspnea can act as an alarm of life-threatening status, the declined perception of dyspnea may result in the delayed seeking of medical care and the severe medical conditions. However, little is known about the actual contribution of the perceptional variability of dyspnea to medical service requirements, expended medical costs, and mortality in the elderly. The objective of this study was to elucidate the impact of perception of dyspnea upon medical care utilization and costs, and mortality in the elderly. For this purpose, we made a survey of Comprehensive Geriatric Assessment (CGA) and the perception of dyspnea in community-dwelling elderly people, and followed up their use of medical care use and cost, and all-cause mortality. Since patients with chronic respiratory diseases complain dyspnea at regular basis, the role of dyspnea as alarm might be obscured. Therefore, we focused on the elderly without chronic respiratory diseases.

We hypothesized that the assessment of the perception of dyspnea in community-dwelling elderly people would identify subjects at risk of greater medical care use and cost, and mortality.

## Materials and Methods

### Study population

The Tsurugaya Project was a community-based Comprehensive Geriatric Assessment (CGA) conducted among elderly Japanese subjects living in Tsurugaya district, a suburban area of Sendai City in Northern Japan, between July 18th and August 8th 2002 (Kuriyama et al., 2006).

At the time of the baseline data collection, 2,780 people aged >70 years old were living in the Tsurugaya district. Letters were sent to all of these people and inviting them to participate in the health survey. Of those invited 1,178 participated in the survey, and 969 gave written informed consent for the CGA survey and medical cost follow-up to be included in the analysis. The study protocol was approved by the institutional review board of the Tohoku University Graduate School of Medicine.

### Questionnaire data

The CGA questionnaire includes (1) demographic characteristics (age, sex, and duration); (2) social factors (visiting friends); (3) lifestyle habits (smoking, alcohol use, and physical activity); (4) physical health (history of chronic medical condition such as stroke or myocardial infection, and present medical condition such as cancer, liver diseases, renal diseases, and angina pectoris).

Data were obtained on (1) body mass index (BMI; in kg/m^2^) as calculated from anthropometric measures by a standardized protocol; (2) the presence or absence of diabetes mellitus, defined as a non-fasting blood glucose concentration >200 mg/dl or the current use of diabetic medication; (3) the presence or absence of hypertension, defined as a self-measured home systolic blood pressure >135 mmHg, a home diastolic blood pressure >85 mmHg, or the use of anti-hypertensive agents; (4) the presence or absence of hypercholesterolemia, defined as a level of 220 mg/dl or over or the current use of lipid-lowering agents; (5) the presence or absence of depressive symptoms, as assessed by using the Japanese versions of the 30-item Geriatric Depression Scale score <11 or >11 (Brink et al., 1982); (6) physical function status, assessed by using the 6-item physical function status measure of the Medical Outcomes Study (MOS) Short-form General Health Survey (Lower MOS scores indicate lower physical function status; Stewart et al., 1988); and (7) leisure-time physical activity, assessed by the frequency and duration of walking, brisk walking, and sports (Niu et al., 2005).

### Spirometry and subjects selection

Spirometry was performed in accordance with the American Thoracic Society recommendations (American Thoracic Society, 1995) in a sitting position with a nose clip by a laboratory technician who did not know the purpose of the study.

**Figure 1:**
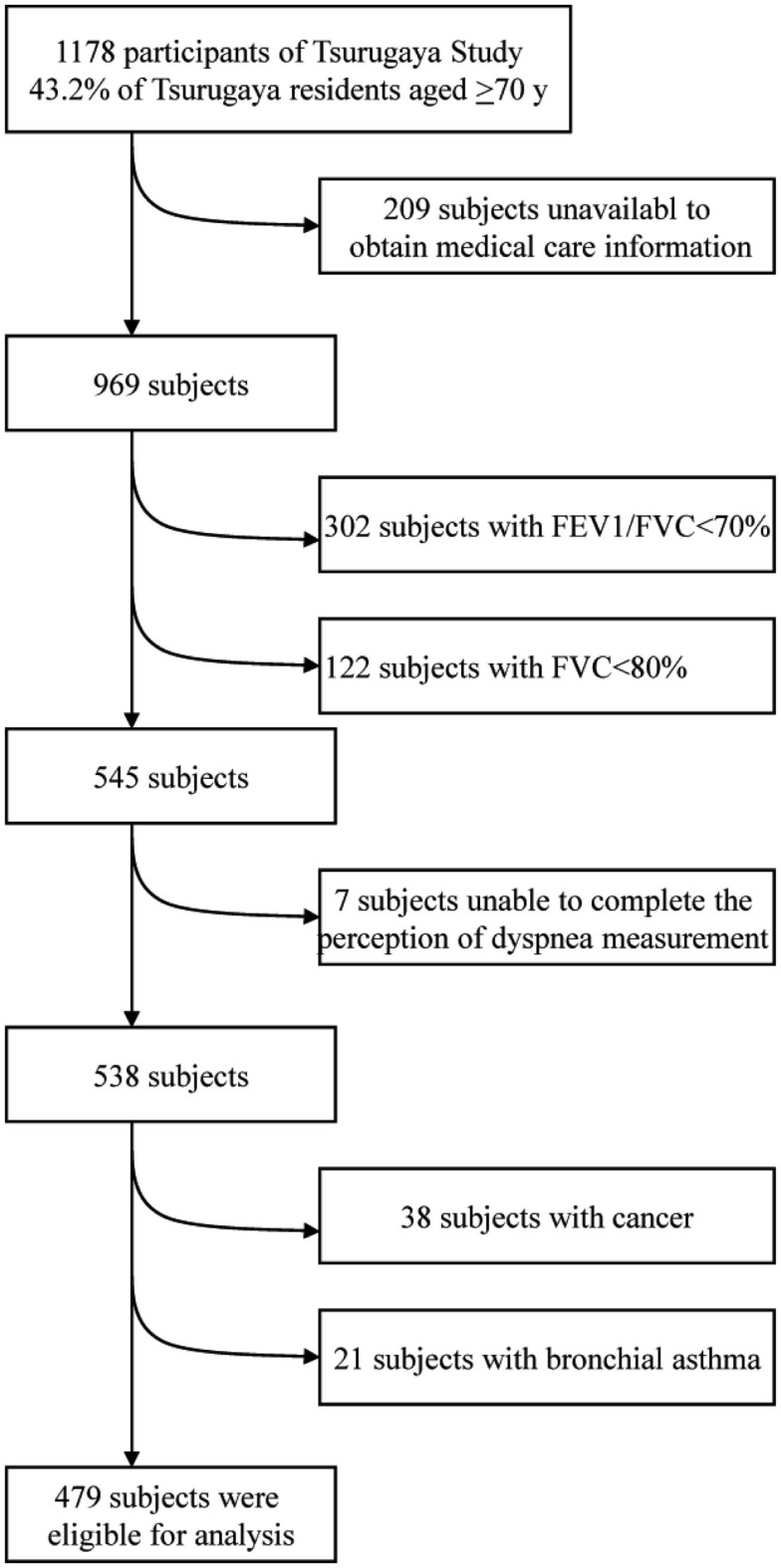
**Exclusion procedure**. FEV1 denotes forced expiratory volume in 1 s, FVC forced vital capacity.

Data about the perception of dyspnea and spirometry with good maneuver were obtained from 969 of the subjects. To exclude the effect of low lung functions such as airway obstructions and pulmonary constrictions on the perception of dyspnea, 424 subjects were excluded whose forced expiratory volume in 1 s (FEV1)/forced vital capacity (FVC) ratio was <70 or whose FVC was <80% predicted. Furthermore, seven subjects were excluded who provided incomplete data of the perception of dyspnea. Finally, 38 subjects with cancer and 21 subjects with bronchial asthma were excluded. Thus, data from 479 subjects was included in the final analyses (Figure 1).

### Perception of dyspnea

Dyspnea was induced by introducing an inspiratory resistive load to the external breathing circuit and was assessed by the modified Borg scale (Kikuchi et al., 1994; American Thoratic Society, 1999). This is well established and validated for measuring physiological as well as psychological sensations, and has been used successfully in and recommended for studies on the perception of dyspnea (American Thoratic Society, 1999; Grant et al., 1999; Lavietes et al., 2000; Livermore et al., 2008).

In brief, the sensation of dyspnea was measured while the subject breathed through the Hans–Rudolph valve with a linear inspiratory resistance (R) of 10, 20, and 30 cmH_2_O/L/s. The loads presented with increasing magnitudes. Neither ventilation nor breathing pattern was controlled during the test. After breathing for 1 min at each level of resistance, the subject rated the sensation of dyspnea (discomfort of breathing) using a modified Borg scale. This is a category scale in which the subject selects a number, from 0 (no dyspnea) to 10 (maximal dyspnea), describing the magnitude of the sensation of dyspnea. The discomfort of breathing was not defined any further, but the subjects were instructed to avoid rating non-respiratory sensations such as headache or irritation of the pharynx.

In order to quantify the perception of dyspnea (ψ) in individuals, the following equation was used (Weiner et al., 2002):

$$\psi =\psi_{\text{R}=10}+\psi_{\text{R}=20}+\psi_{\text{R}=30}-3\times \psi_{\text{R}=0}$$

where ψ_R=0, 10, 20, 30_ represents the Borg score at *R* = 0, 10, 20, 30 cmH_2_O/L/s, respectively. Here we tried to eliminate the mouth piece effect by subtracting the baseline sensation.

We further classified the perception of dyspnea into tertiles according to the level of ψ such as low (0 ≤ ψ < 3, *n* = 159), intermediate (3 ≤ ψ > 9, *n* = 166), and high (ψ ≥ 9, *n* = 175) groups.

### Outcome measurements

We collected prospective data on medical care utilization and costs, and death for each subject from August 2002 to March 2008. The health insurance system, which is compulsory for everyone living in Japan and covers almost all medical treatment and fees for medical providers, has two classifications (Tsuji et al., 1999). One is the insurance system for employes and their dependents, and the other is a system of community-based health insurance for retired persons, pensioners, the self-employed, and their dependents. The latter system is called the National Health Insurance (NHI) plan and covers almost all elderly people with very few exceptions. Therefore, we obtained allowable charges from the NHI Claim History files of the Miyagi NHI association by assessing NHI payments (reimbursement), beneficiary copayments and deductible charges by obtaining allowable charges.

Claim files included the beneficiary’s ID number, personal name, the code number of the medical provider, number of visits for out-patient care, number of days for in-patient care, the death day if they died, and the charges for out-patient and in-patient care, respectively. In order to know the cause of death, we found the hospital where they were finally involved. If the hospital became bankrupt or an uncertain medical course was described in the medical chart, the cause of death was unknown.

### Statistical analysis

The subjects’ baseline characteristics are presented according to the perception of dyspnea categorized into tertiles as the independent variables after adjustment for age and sex (Table 2). Differences in variables among the categories were examined by analysis of covariance for linear trends using the median values of the perception of the dyspnea in each category.

We calculated medical care costs and medical care use as per month values. Per month values for each subject were calculated by dividing the accumulated values through follow-up by the number of months observed. Except for the data shown in Figure 3, we examined per month values in order to avoid underestimating the medical care use and costs of the subjects who died or emigrated. The monetary values were converted into US dollars using the rate of 1 US$ = ¥115.

The impact of the perception of dyspnea upon the medical costs, and the number of out-patient visits and hospitalized days were respectively investigated, using analysis of covariance (Table 3). In these analyses, we regarded the following data as covariates for multivariate analysis: age category (70–74, 75–79, 80–84 years); sex; BMI (continuous variable); hypertension, hypercholesterolemia, diabetes mellitus, history of stroke, myocardial infarction, or angina pectoris, liver diseases, renal diseases (presence or absence); cognitive function (mini-Mental State Examination score of <24 or ≥24); depressive symptoms (Geriatric Depression Scale scores of <11 or ≥11); smoking (never, former, and current smoker); use of alcohol (never, former, and currently drinking); use of tranquilizer (yes or no); physical functioning status (MOS scores [continuous variables]); leisure-time physical activity (Level 1: no sports, no brisk walking, no walking; Level 2: no sports, no brisk walking; low amount of walking; Level 3: no sports, no brisk walking, high amount of walking; Level 4: no sports, low amount of brisk walking, any amount of walking; Level 5: no sports, high amount of brisk walking, any amount of walking; Level 6: any amount of sports, any amounts of brisk walking, any amount of walking). Differences in variables among the categories were examined for a linear trend using the median values of the perception of the dyspnea in each category.

The survival curves according to the categories of dyspnea sensation were displayed using the Kaplan–Meier curves and also estimated by the log-rank test. We used Cox proportional hazard models to compare the all-cause mortality among the groups categorized by the perception of dyspnea in multivariate analysis, expressed by the hazard ratio. We ascertained that the proportional-hazards assumption was not violated by using log–log plots, i.e., −ln{−ln(survival)} curves versus ln(analysis time) of survival curves of the 3 categories by the perception of dyspnea, to check that curves were parallel.

A significant difference was defined as *p* < 0.05. All statistical analyses were performed by using the Statistical Analysis System 9.1 edition for Windows (SAS Institute Inc., Cary, NC, USA).

## Results

**Figure 2:**
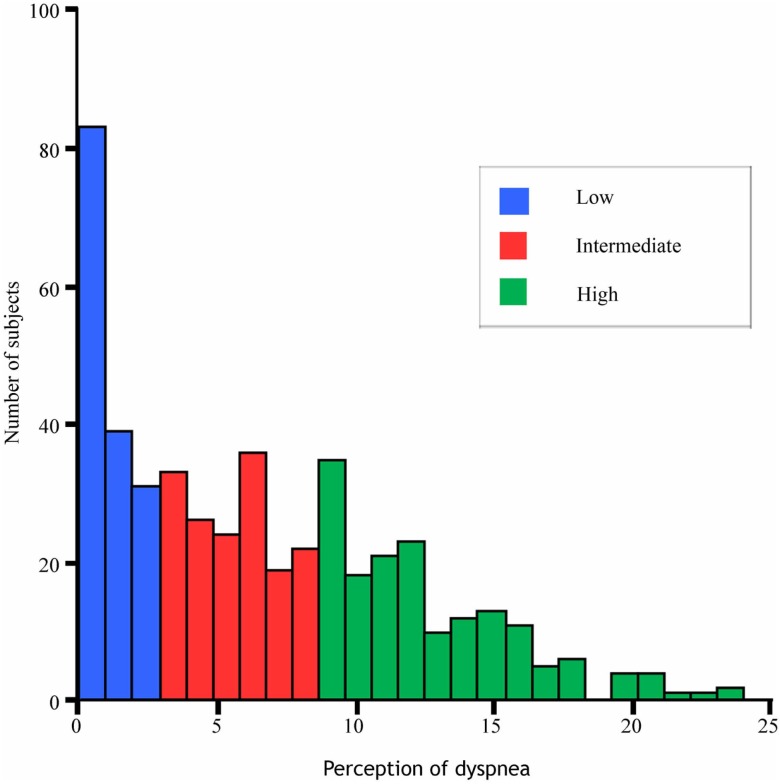
**Distributions of the perception of dyspnea**.

After exclusion (Figure 1), 479 subjects attributed to the final analysis. The distribution of the perceptions of dyspnea, which did not follow the Gaussian distribution, is shown in Figure 2. The subjects’ baseline characteristics according to categories of the perception of dyspnea are shown in Table 1. No apparent associations were observed among BMI, hypertension, hypercholesterolemia, diabetes mellitus, coronary heart disease, stroke, liver and renal diseases, depressive state, drinking, and smoking status.

**Table 1:** **Baseline characteristics according to dyspnea tertile (*n* = 479)**.

	All	Tertile of dyspnea			*p* for trend
		Low	Intermediate	High	
No. of participants	479	153	160	166	–
Age 70–74 (%)	42.8	38.6	40.6	48.8	0.08
Age 75–79 (%)	34.7	33.3	40.0	30.7	0.63
Age 80–84 (%)	14.6	16.3	14.4	13.3	0.48
Age ≥ 85 (%)	7.9	11.8	5.0	7.2	0.15
Sex (male, %)	36.5	25.5	35.6	47.6	<0.0001
BMI (kg/m^2^)	23.9 (23.6–24.2)	24.9 (24.0–25.8)	24.5 (23.7–25.3)	24.5 (23.7–25.3)	0.28
Hypertension (%)	66.0	66.7	68.8	62.7	0.62
HCL (%)	45.5	48.4	45.0	43.4	0.95
Diabetes (%)	10.4	14.4	6.3	10.8	0.46
MMSE ≥ 24 (%)	93.1	90.9	95.0	93.4	0.76
**SMOKER**					
Current smoker (%)	9.0	6.5	10.6	9.6	0.87
Ex-smoker (%)	25.1	19.6	24.4	30.7	0.87
Never-smoker (%)	63.3	70.6	64.4	55.4	0.67
**DRINKING**					
Current drinking (%)	9.2	9.2	9.4	9.0	0.46
Ex-drinking (%)	45.9	55.6	45.6	37.4	0.13
Never-drinking (%)	40.1	28.1	44.4	47.0	0.08
**SELF-REPORTED ILLNESS**					
Renal (%)	6.5	3.3	7.5	8.4	0.08
CHD (%)	9.8	7.2	13.8	8.4	0.42
Stroke (%)	5.0	5.2	3.8	6.0	0.90
Liver (%)	6.9	5.9	8.1	6.6	0.97

*BMI, body mass index; HCL, hypercholesterolemia; MMSE, mini-Mental State Examination, CHD, coronary heart disease*.

*Variables are expressed as mean (95% CI) or %*.

Among 479 subjects with normal lung function, we compared the medical care utilization and its cost according to the perceptions of dyspnea (Table 2). We analyzed the crude data, the data after adjustment for age, sex, and BMI, and the data adjusted for a variety of confounders. A statistically significant inverse association was observed between the perception of dyspnea and the hospitalized days per month in the crude analysis (*p* = 0.02). This association became more significant after adjustment for age, sex, and BMI, and multivariate analysis (*p* = 0.01 and *p* < 0.01, respectively). Although medical care costs for in-patient care was not significantly different in the crude comparison, after adjustment for age, sex, and BMI, and for multivariate, there was a significant reverse association between the perception of the dyspnea and the medical care cost (*p* = 0.03 and 0.02, respectively).

**Table 2:** **Adjusted medical use and costs in relation to dyspnea tertile (*n* = 479)**.

	Tertile of dyspnea			*p* for trend
	Low	Intermediate	High	
No. of participants	153	160	166	
**IN-PATIENT CARE**				
No. of hospital days (days/month)				
Crude	0.81 (0.49–1.13)	0.70 (0.39–1.02)	0.30 (−0.01–0.60)	0.02
Adjusted for age, sex, and BMI	0.61, (0.07–1.16)	0.51, (0.00–1.02)	0.03, (−0.48–0.54)*	0.01
Multivariate^#^	1.72, (0.54–2.91)	1.73, (0.58–2.89)	1.12, (−0.05–2.29)*^†^	<0.01
Medical cost (dollars/month)
Crude	184.8, (119.3–250.3)	180.9, (116.8–245.0)	114.0, (51.1–176.9)	0.13
Adjusted for age, sex, and BMI	194.4, (82.7–306.0)	181.6, (78.6–284.7)	93.7, (−10.8–198.2)	0.03
Multivariate^#^	587.2, (335.9–838.6)	592.7, (347–838.4)	480.6, (232.2–728.9)*^†^	0.02
**OUT-PATIENT CARE**				
No. of hospital visits (days/month)				
Crude	5.29, (4.53–6.06)	5.2, 5(4.51–5.98)	6.00, (5.29–6.71)	0.18
Adjusted for age, sex, and BMI	5.21, (3.94–6.48)	5.21, (4.03–6.38)	5.96, (4.77–7.15)	0.16
Multivariate^#^	8.57, (5.75–11.39)	8.40, (5.65–11.14)	9.26, (6.48–12.04)	0.20
Medical cost (dollars/month)
Crude	284.8, (246.0–323.5)	268.4, (230.5–306.3)	292.4, (255.2–329.6)	0.78
Adjusted for age, sex, and BMI	282.6, (215.6–349.6)	267.0, (205.2–328.8)	291.6, (228.9–354.3)	0.75
Multivariate^#^	472.1, (325.3–618.9)	447.8, (304.3–591.3)	468.1, (323.1–613.2)	0.88
Total medical cost (dollars/month)
Crude	469.6, (390.2–548.9)	449.3, (371.6–526.9)	406.4, (330.2–482.6)	0.26
Adjusted for age, sex, and BMI	476.9, (341.2–612.7)	448.6, (323.3–573.9)	385.3, (258.3–512.3)	0.11
Multivariate^#^	1059.3, (763.8–1354.8)	1040.5, (751.7–1329.3)	948.7, (656.8–1240.7)	0.04

*^#^Adjusted for age (70–74, 75–79, 80–84, and 85+ years), sex, body mass index, hypertension, hypercholesterolemia, diabetes, history of coronary heart disease, history of stroke, history of renal disease, history of hepatic disease, drinking status, smoking status, physical activity, physical performance, depressive symptoms, Mini-Mental State Examination, and use of tranquilizers*.

*Variables are presented as mean (95% CI)*.

*p Values for trend are based on median levels in each tertile*.

*p < .05 as compared with “Low” group (Tukey’s post hoc).

*^†^p < .05 as compared with “Intermediate” group (Tukey’s post hoc)*.

In out-patient care, both the number of medical service visits and medical care costs did not significantly differ among the categories of the perception of dyspnea. These results were not changed after the adjustment for age, sex, and BMI, and the adjustment for all cofounders. Eventually, although the total medical care costs was not different in crude comparison, after adjustment for the confounders, there was a significant reverse association between the perception of the dyspnea and the total medical care cost (*p* = 0.04).

**Figure 3:**
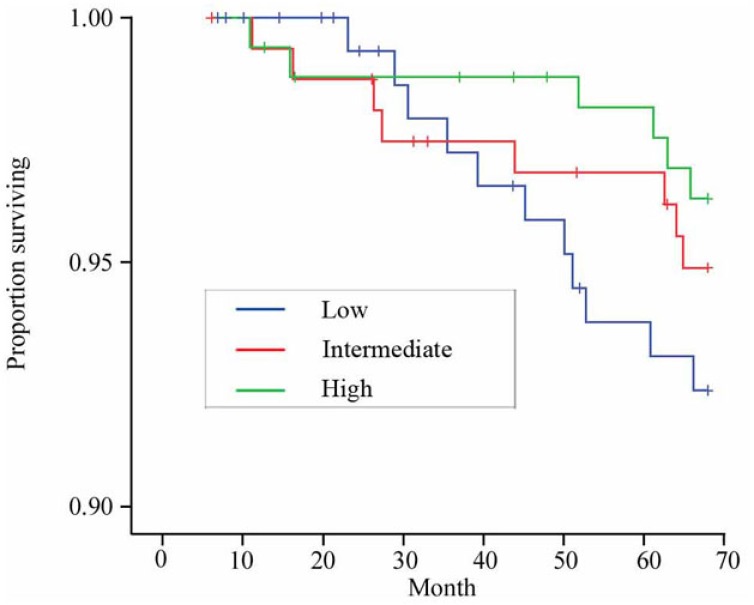
**Kaplan–Meier curves for survival of subjects according to the perception of dyspnea**.

The Kaplan–Meier survival curves and their relation to the perception of dyspnea are shown in Figure 3. Statistics using the long-rank test did not show a significant difference among the groups (*p* = 0.26). The results of Cox regression analysis for the relationship between the perception of dyspnea and mortality are shown in Table 3. There was a significant reverse association between the perception of the dyspnea and hazard ratio of mortality (*p* = 0.04). The high perception of dyspnea group showed significantly lower hazard ratio than the low perception of dyspnea group. Among the 11 deaths in the low perception group, 2 died of pancreas cancer, 2 of heart failure, 1 of aortic aneurysm, 1 of ischemic heart disease, 1 of interstitial pneumonia, 1 of pneumonia and 1 of diabetes mellitus. Among the 8 deaths of intermediate perception group, 2 died of stroke, 2 of lung cancer, 1 of heart failure, 1 of burn, and 2 for unknown causes. Among the 6 deaths of high perception group, 1 died of lung cancer, 1 of pancreas cancer, 1 of respiratory failure, 1 of suffocation, and 2 for unknown causes.

**Table 3:** **Hazard ratios of all-cause mortality according to dyspnea tertile (*n* = 479)**.

	Tertile of dyspnea			*p* for trend
	Low *n* = 153	Intermediate *n* = 160	High *n* = 166	
Person-months of follow up	9684	10452	10963	–
No. of cases	11	8	6	–
Hazard Ratio (95% Confidence Interval)
Crude	1.00	0.67, (0.27–1.66)	0.48, (0.18–1.29)	0.14
Adjusted for age, sex, and BMI	1.00	0.72, (0.28–1.82)	0.48, (0.17–1.31)	0.15
Multivariate^#^	1.00	0.65, (0.23–1.89)	0.31, (0.10–0.97)	0.04

*^#^Adjusted for age (70–74, 75–79, 80–84, and 85+ years), sex, body mass index, hypertension, hypercholesterolemia, diabetes, history of coronary heart disease, history of stroke, history of nephropathy, history of hepatic disease, drinking status, smoking status, physical activity, physical performance, depressive symptoms, Mini Mental State Examination, and use of tranquilizers*.

**p* Values for trend are based on median levels in each quartile*.

## Discussion

This is the first community-based screening of the perception of dyspnea. Our study showed that the blunted perception of dyspnea could be related to future greater medical care use and costs, especially in the medical care cost for in-patient care. Moreover, we also showed that the blunted perception of dyspnea could be related to future greater mortality in community-dwelling elderly people. These results suggest that subjects maintaining a perception of dyspnea did not develop a more severe state of diseases and did not require higher rates of admission to the hospital than subjects with a blunted perception of dyspnea. The awareness that early events portend an increasingly severe condition is important for timely treatment. Since dyspnea serves as an alarming symptom harboring severe pathology, patients with a well-maintained perception of dyspnea have the potential to seek care earlier before the stages which require intensive care. On the other hand, the elderly with a blunted perception of dyspnea might be high risk group for future greater medical care use presumably due to the severe diseases and risk of death.

Previous studies concerning the blunted perception of dyspnea mainly focused on asthmatics. Kikuchi et al. (1994) reported a significantly decreased perception of dyspnea against inspiratory resistive load and a decreased hypoxic ventilatory response in patients with near-fatal asthma. The impaired sensitivity to dyspnea in patients with near-fatal asthma was also confirmed by blunted dyspnea at peak exercise and breath holding (Berreiro et al., 2004; Nannini et al., 2007). Moreover, Magadle et al. (2002) prospectively confirmed that asthmatic subjects with a low perception of dyspnea are at high risk of hospitalization for near-fatal and fatal asthma. All of these findings pointed to perception as being a key factor in the overall management of asthma. However, the role of dyspnea perception has heretofore not been evaluated in the respect of the health management of elderly people.

In this study, elderly women were significantly more likely (*p* < 0.001) to have a blunted dyspnea perception than were elderly men (Table 2). In asthmatics, several studies have shown that females report more dyspnea than males (Magadle et al., 2002; Weiner et al., 2002), and others have not (Bijl-Hofland et al., 1999; Stravinskaite et al., 2005). However, there is no report describing the gender difference in the perception of dyspnea in the non-asthmatic subjects over 70 years old. In pain perception, although the fact that females display a greater sensitivity to pain than males is well established, it was reported that the difference decreased with age and became not significant (Pickering et al., 2002). Despite many hypotheses such as biological, hormonal, genetic, that have been proposed, the cause of gender difference in pain perception still remains unclear. Cultural factors have also been adduced, with the impact of education leading to a stoic attitude and an under-reporting of pain in males (Otto and Dougher, 1985). Traditional Japanese women are educated to be patient by their mother-in-law once they get married (Yamamoto and Wallhagen, 1998). This tendency is more prominent in the north part of Japan where our study was conducted. Since our subjects were enough old to be traditional, this could be a reason why the perception of dyspnea in female scores were smaller than male in our subjects.

Like any other unpleasant alarming sensation, the impaired perception of dyspnea occurs with aging (Tack et al., 1982). In nociception, electrophysical measures have found lower nerve conduction velocity in older individuals, suggesting impairment in axon structure and function (Bouche et al., 1993). However, the cellular and molecular basis of why nociception is altered with age still remain unclear. In the elderly, there is perceptual slowing both at the peripheral and central stages of processing (Walsh et al., 1979). Further studies are required to elucidate the possible involvement of both central and peripheral processing dysfunction in the age-related impairment of dyspnea perception.

Dyspnea in the elderly can be caused by many conditions, with cardiac and pulmonary causes being the most common (Pederden et al., 2005). The most serious limitation of this study is the unavailability of the diagnosis for each instance of medical care use. This prevents us from investigating the effects of the perception of dyspnea on a particular disease. However, it is often very difficult to clinically determine the cause of dyspnea, especially in the elderly (Cabanesb et al., 2001).

In conclusion, this is the first study to investigate the effect of the perception of the dyspnea, on medical care use and costs, and all-cause mortality in community-dwelling elderly people. Our study shows that the blunted perception of dyspnea could be related to future greater medical care costs, especially in the medical care cost for in-patient care. Moreover, it is related to higher mortality. Therefore, the screening of dyspnea perception in the elderly as part of routine medical care may be worthwhile, as blunted dyspnea perception may possibly indicate the need for more careful subsequent medical follow-up.

## Conflict of Interest Statement

The authors declare that the research was conducted in the absence of any commercial or financial relationships that could be construed as a potential conflict of interest.
